# Endoscopic submucosal dissection for laterally spreading tumor inside gallbladder: A novel organ preserving option

**DOI:** 10.1055/a-2067-4497

**Published:** 2023-04-21

**Authors:** Saif Ullah, Dan Liu, Bing-Rong Liu

**Affiliations:** 1 Department of Gastroenterology, The First Affiliated Hospital of Zhengzhou University, Zhengzhou, China; 2 State Key Laboratory of Esophageal Cancer Prevention and Treatment, Zhengzhou University, Zhengzhou, China

A 61-year-old man was admitted for management of gallbladder polyps. The patient refused cholecystectomy as he wished to retain his gallbladder, but consented to undergo pure flexible endoscopic gallbladder-preserving polypectomy.

With the patient under general anesthesia, a colonoscope was advanced into the colon for colonic cleansing, following which a detachable colonic exclusion balloon was placed in the transverse colon and inflated to 3.0–3.5 cm in diameter to occlude the transverse colonic lumen. Disinfection of the distal colon was then completed with a 0.1 % povidone–iodine solution ( Fig. 1 ). A disinfected colonoscope with a transparent cap attached on its tip was inserted and an incision was made on the right anterior wall of the rectum using a HookKnife and IT Knife. The endoscope was passed through the incision and advanced upward until the gallbladder was identified. A 1.5-cm full-thickness longitudinal incision was created in the gallbladder wall, and the tip of the endoscope was inserted into the gallbladder cavity. Several small superficial polyps were seen and resected using an electrocoagulation snare ( Fig. 2 a, b ). This left one invasive, deep, and large (1 × 0.8 × 0.3 cm) pedunculated polyp, which was removed by endoscopic submucosal dissection ( Video 1 ). The technique used included a circular incision ( Fig. 2 c ) around the lesion using the HookKnife and then resection using a snare ( Fig. 2 d ). After the resection had been completed ( Fig. 2 e ), the gallbladder and rectal incisions were closed with endoclips ( Fig. 2 f ).

**Fig. 1:**
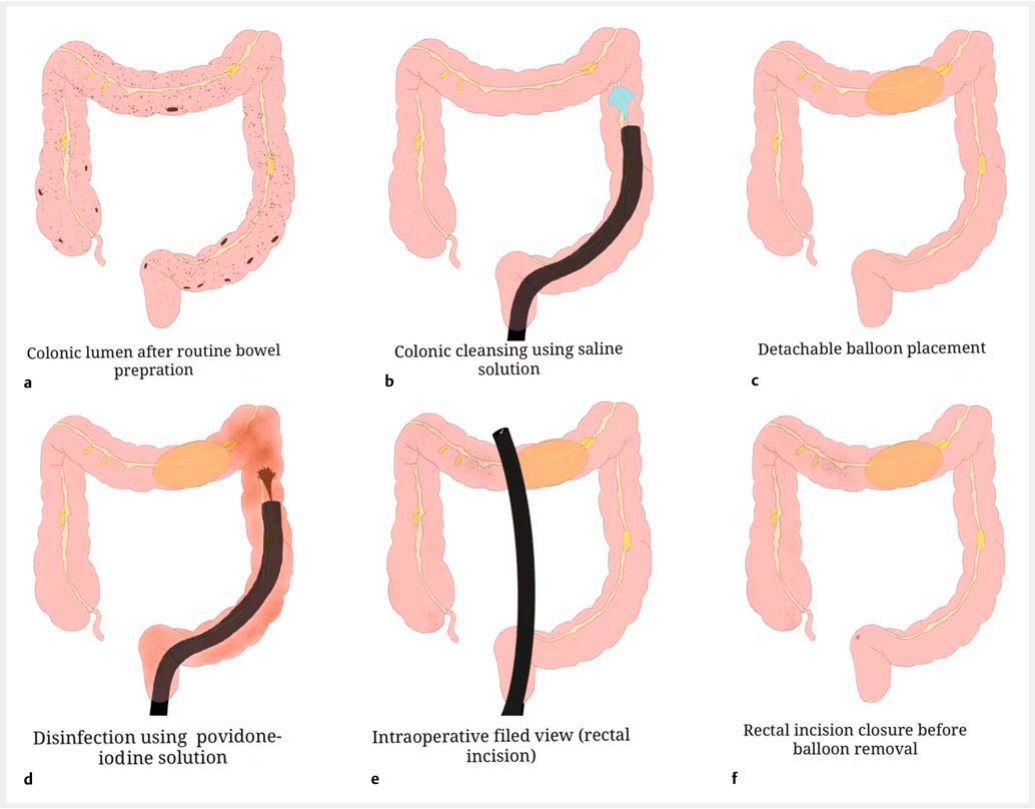
Schematic diagram showing placement of a detachable balloon in the transverse colon and disinfection of the distal colonic and rectal lumen.

**Fig. 2:**
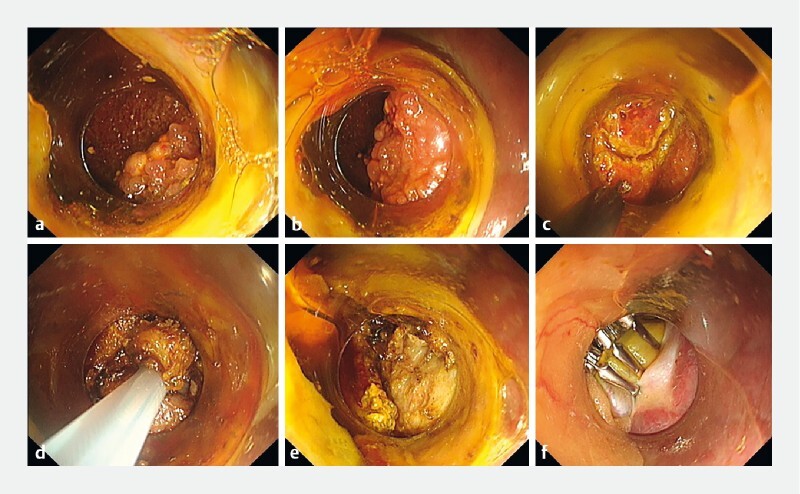
Endoscopic images showing: **a, b** multiple gallbladder lesions (polyps); **c** a circular incision made around the remaining large lesion using a HookKnife; **d** resection and removal of the lesion using a snare; **e** the appearance after lesion resection; **f** closure of the gallbladder incision using endoclips.

**Video 1** Pure flexible endoscopic gallbladder-preserving polypectomy, including endoscopic submucosal dissection of the largest polyp, is performed to manage gallbladder polyps.


The tissue specimen was sent for histopathology and showed cholesterol polyps with chronic hyperplasic fibrous connective tissue inflammation. The patient received levofloxacin (0.5 g four times daily) and cefoperazone–sulbactam (2 g four times daily) intravenously for 3 days after the procedure. He was discharged on day 4 and has remained well during 15 months of follow-up.

This case illustrates that gallbladder-preserving polypectomy may be a feasible and minimally invasive technique for the management of patients with large gallbladder polyps. Although this procedure is unlikely to replace cholecystectomy, it could prove useful for patients wanting to avoid surgical resection.

Endoscopy_UCTN_Code_TTT_1AT_2AB

## Notes

Endoscopy E-Videos

https://eref.thieme.de/e-videos
*E-Videos* is an open access online section of the journal *Endoscopy* , reporting on interesting cases and new techniques in gastroenterological endoscopy. All papers include a high-quality video and are published with a Creative Commons CC-BY license. Endoscopy E-Videos qualify for HINARI discounts and waivers and eligibility is automatically checked during the submission process. We grant 100% waivers to articles whose corresponding authors are based in Group A countries and 50% waivers to those who are based in Group B countries as classified by Research4Life (see: https://www.research4life.org/access/eligibility/ ). This section has its own submission website at https://mc.manuscriptcentral.com/e-videos

